# An Efficient Biometric-Based Algorithm Using Heart Rate Variability for Securing Body Sensor Networks

**DOI:** 10.3390/s150715067

**Published:** 2015-06-26

**Authors:** Sandeep Pirbhulal, Heye Zhang, Subhas Chandra Mukhopadhyay, Chunyue Li, Yumei Wang, Guanglin Li, Wanqing Wu, Yuan-Ting Zhang

**Affiliations:** 1 Institute of Biomedical and Health Engineering, Shenzhen Institutes of Advanced Technology, Shenzhen 518055, China; E-Mails: sandeep@siat.ac.cn (S.P.); hy.zhang@siat.ac.cn (H.Z.); cy.li1@siat.ac.cn (C.L.); gl.li@siat.ac.cn (G.L.); ytzhang@ee.cuhk.edu.hk (Y.-T.Z.); 2 Key Laboratory for Health Informatics of the Chinese Academy of Sciences (HICAS), Shenzhen Institutes of Advanced Technology, 1068 Xueyuan Avenue, Shenzhen University Town, Shenzhen 518055, China; 3 School of Engineering and Advanced Technology, Massey University, Palmerston North 4442, New Zealand; E-Mail: S.C.Mukhopadhyay@massey.ac.nz; 4 Shenzhen Nanshan District Xili Hospital, Shenzhen 518055, China; E-Mail: fox_gxh@sina.com; 5 Key Laboratory of Human-Machine-Intelligence Synergic System, Shenzhen Institutes of Advanced Technology, Chinese Academy of Sciences (CAS), Shenzhen 518055, Guangdong, China; 6 Joint Research Centre for Biomedical Engineering, Chinese University of Hong Kong, Shatin N.T., Hong Kong, China

**Keywords:** Body Sensor Network (BSN), biometric, efficiency, Electrocardiogram (ECG), Heart Rate Variability (HRV), security

## Abstract

Body Sensor Network (BSN) is a network of several associated sensor nodes on, inside or around the human body to monitor vital signals, such as, Electroencephalogram (EEG), Photoplethysmography (PPG), Electrocardiogram (ECG), *etc.* Each sensor node in BSN delivers major information; therefore, it is very significant to provide data confidentiality and security. All existing approaches to secure BSN are based on complex cryptographic key generation procedures, which not only demands high resource utilization and computation time, but also consumes large amount of energy, power and memory during data transmission. However, it is indispensable to put forward energy efficient and computationally less complex authentication technique for BSN. In this paper, a novel biometric-based algorithm is proposed, which utilizes Heart Rate Variability (HRV) for simple key generation process to secure BSN. Our proposed algorithm is compared with three data authentication techniques, namely Physiological Signal based Key Agreement (PSKA), Data Encryption Standard (DES) and Rivest Shamir Adleman (RSA). Simulation is performed in Matlab and results suggest that proposed algorithm is quite efficient in terms of transmission time utilization, average remaining energy and total power consumption.

## 1. Introduction

**Figure 1:**
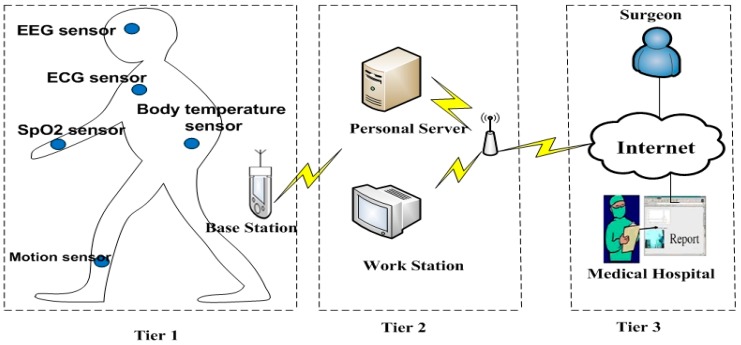
Body Sensor Network Architecture, with the permission form [3].

Wireless Body Area Network (WBAN) or Body Sensor Network (BSN) is a very important application of Wireless Sensor Networks (WSNs) for healthcare monitoring, which takes up a wireless network of different in-body, on-body and around-body attached sensor nodes. The general BSN architecture is demonstrated in Figure 1. Though BSNs are the best method for telemedicine, sensor nodes carry very vital information, thus privacy and security become very important in these networks. In view of the fact that each person exhibits different physiological behaviors, the human body itself can be used as the best source to implement security. It would be rather difficult for an attacker to hack the data, as discussed in Wang *et al.* [1]. Biometric technology is the automatic detection of behavioral or physiological traits of the human body. The ECG is a unique individual trait and it satisfies ideal biometric characteristics [2], as its properties completely depend on the human body and its heart beats. Therefore, we used Electrocardiogram (ECG) as a biometric characteristic in our research to secure BSN.

In BSN, many security methods [4] are proposed using symmetric as well as asymmetric key-based approaches to obtain privacy, reliability and accuracy during data transmission. In [5], a symmetric key-based approach is used to implement security in data transmission for BSN. Though, only one private key is used in whole BSN, if any sensor node is compromised then private information will no longer be secure, as presented in William *et al.* [6]. Whereas, asymmetric techniques are resource constrained, as explained in Poon *et al.* [2]. Hence, these are not cost-efficient and feasible solutions for securing BSN. Some researchers use a combination of both symmetric and asymmetric techniques to increase the security and privacy in BSN [4]. Nonetheless, all these conventional approaches are based on external keys for data authentication in BSN. The biometric based security increases reliability, provides rapid action and cost efficient security compared to conventional cryptographic key-based techniques. Therefore, in modern research, biometric characteristics are employed for implementing data authentication in WBAN, as studied in [7,8,9,10,11]. Miao *et al.* [12] used ECG as biometric trait and AES (Advanced Encryption standard) is applied for data integrity and data authentication between source and destination. The Physiological Signal based Key Agreement (PSKA) scheme, proposed in Venkat *et al.* [13], uses Fuzzy vault logic between source and destination for key synchronization. There are two problems associated with that technique: (i) if key size is small, hackers can guess key by brute force attacks; and (ii) if key size is large, destination will require high computational cost. Yao *et al.*, based on ECG, developed a model to achieve data integrity and data confidentiality in BSN [14]. Bao *et al.* [15] uses Inter-pulse intervals (IPI) from ECG and Photoplethysmogram (PPG) to accomplish security in BSN. Even though their research claims accurate security model for BSN, it does not provide cost effective and energy efficient system for data authentication in BSN. The IPI from PPG is used to generate distinctive keys in Zhang *et al.* [16]. The matchless and difficult algorithm used for key generation guarantees accurate authentication. However, high cost and excessive amount of power are required to implement this technique in BSN. Ramli *et al.* [17], suggests a biometric based model; which uses Message Authentication Protocol (MAC) as a key for authentication between source and destination and omits complex key generation methods for implementing authentication in BSN. Their research does not discuss energy efficiency as well as power efficiency, and also no specific authentication protocol is suggested.

One of the major problems in all aforementioned research is the utilization of complex key generation mechanism based on different security techniques, algorithms and models. As complex and time consuming procedures are used, cost effective and energy efficient security solution for BSN was not achieved. To remedy these problems, we propose a novel algorithm based on Heart Rate Variability (HRV). The authentication protocol in the proposed algorithm is the logical exclusive-OR (X-OR) operation between Standard Deviation of NN interval (SDNN) and Root-Mean Squared of the Successive Differences (RMSSD) ratio, age and gender information of the source. The NN interval is the time duration between two successive R-R peaks in ECG waveform. The output of X-OR is a 16-bit binary number, which is acting as the authentication key between source and destination to implement security during data transmission in BSN. However, for additional security, SHA-1 hashing algorithm is used to hide original message before its transmission. Our research aims to signify the utilization of HRV for simple key generation procedure in order to provide efficient method for securing Body Sensor Networks (BSN). The suggested method is cost efficient (by offering low resources utilization), time efficient (by requiring less transmission time for sending information as a simple key generation procedure is used), and energy and power efficient (by demanding less energy and power consumption) to secure BSN. The proposed algorithm is compared with Physiological Signal based Key Agreement (PSKA), Data Encryption Standard (DES) and Rivest Shamir Adleman (RSA) and it provides more efficient security than all the compared techniques. The key point of our research lies in the fact that it eradicates complex key generation procedures and presents simple and efficient key generation mechanism based on ratio between SDNN and RMSSD for data authentication in BSN.

The rest of the paper is organized as follows. In Section 2, methodology of proposed algorithm is discussed. The Section 3 includes experiment setup followed by simulation and results in Section 4. Finally, the paper is concluded in Section 5.

## 2. Method and Implementation

### 2.1. Wearable System for Physiological Signal Collection

**Figure 2:**
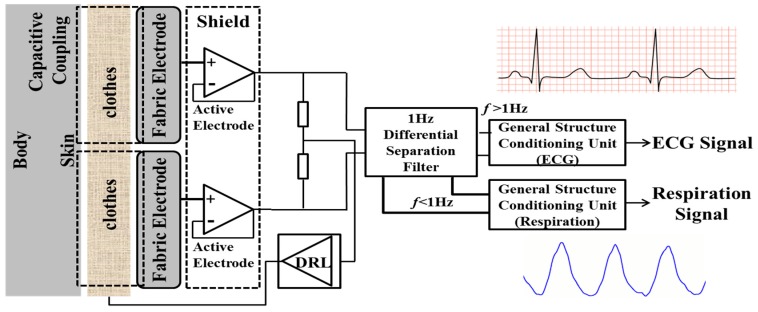
Block diagram of capacitive measurement system.

We design a device for extraction of ECG signal from a textile electrode. This device is based on filters and amplifiers for measuring ECG and breathing signal, its block diagram is shown in Figure 2. For sensing breathing activity and ECG signal, differential separation filter and a common part were included in the device, respectively. The common part consisted of two buffers, to provide impedance to the capacitive coupling with low impedance entailed by the consequent circuitry. Our study utilized operational amplifier ICs having high input resistance. The differential separation filter split the input signal into low frequency component including breathing signal (<1 Hz) and high frequency component containing ECG signal (>1 Hz). The circuit diagram from [18], was used in order to reduce common mode noise mainly due to power line interference. The differential separation filter divides the input signal into sets of subtractors, amplifiers and integrators according to DC suppression circuit. The part for sensing ECG signal consisted of an instrumentation amplifier, a high-pass filter (HPF), a low pass filter (LPF) and two inverting amplifiers. The circuit elements of the LPF and the HPF were designed to achieve a cutoff frequency of 40 and 5 Hz, respectively.

Software platform is responsible for extracting bio-information from raw data and for calculating the Heart Rate Variability (HRV) of physiological signals, which is further used for securing BSN. The bio-signals are generally weak and easily damaged by different kinds of noise such as instrumentation noise, power line interference, electrosurgical noise, motion artifacts, baseline drift, electrode contact noise, and other less considerable noise sources, which cannot be filtered entirely by using only hardware platform. Therefore, a finite impulse response (FIR) band-pass filter for correcting baseline stroll; a digital 60-Hz notch filter for reducing the power line interference; a multi-scale mathematical morphology (3 M) filter for removing motion artifacts and power line interference; and a differential operation for smoothing and normalizing have been integrated into the software. In order to measure the HRV, an adaptive QRS detect algorithm, which was easy to implement on a simple, real-time device developed by our laboratory in a previous study, has been adopted to extract RR interval series for HRV analysis; with 99.3% detection rate [19]. The calculated time domain approaches SDNN and RMSSD of HRV are obtained according to the standards of measurement, proposed by the Task Force of the European Society of Cardiology and the North American Society of Pacing and Electrophysiology, which describes the detail of physiological correlates of HRV and calculation methods.

### 2.2. Proposed Algorithm Model

**Figure 3:**
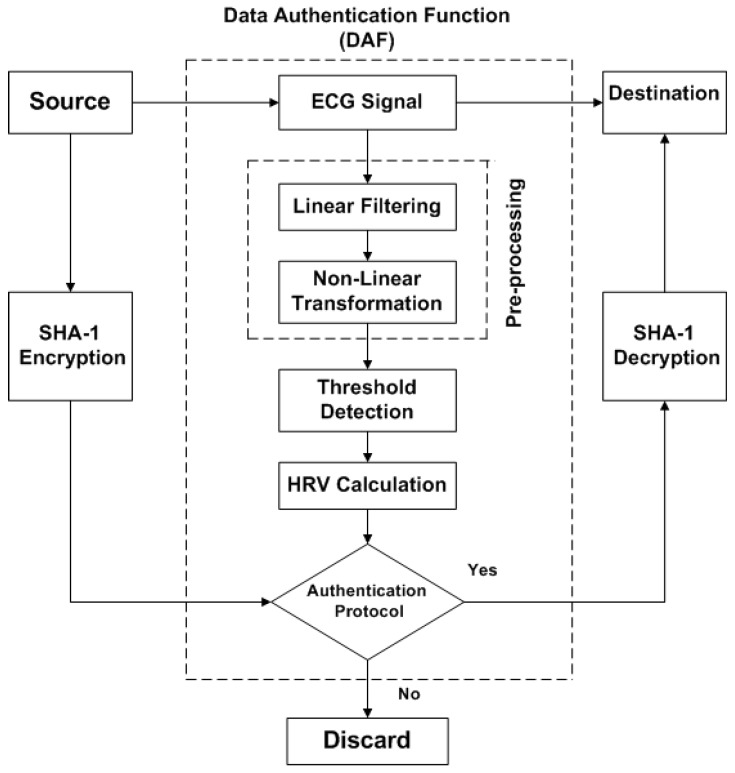
Block diagram of Biometric-based Proposed Algorithm.

Our proposed algorithm is simple because it eliminates the use of complex key generation procedures. However, in BSN, those techniques, which are based on intricate key generation procedures, require high computational cost for management of keys as well as consume a lot of time, energy and power during data transmission. The block diagram of our proposed algorithm is shown in Figure 3. The output of authentication protocol in Data Authentication Function (DAF) is acting as a key. Once this key matches, then the generated message from the source can be transmitted to destination. In case the receiver does not match statistically, transmission will not be started and the message will be discarded, as demonstrated in Figure 3. Even though DAF is used, data reliability and accuracy can be achieved. But, to increase the level of security, SHA-1 hashing scheme is employed for encryption of the original message. This hashing technique is very simple, easily applied and less complex. Thus, it provides low cost encryption.

The DAF includes four main parts; (i) pre-processing of physiological signal ECG, which is combination of linear filters (step 1) and non-linear transformation (step 2); (ii) threshold detection for QRS complex (step 3); (iii) HRV calculation (step 4); and (iv) authentication protocol (step 5). ECG is used as biometric trait for data authentication in our proposed algorithm. The details of step 1 to step 5 have been illustrated in Section 2.3. Basically, ECG waveform contains five different deflections, namely P, Q, R, S and T. QRS-complex contains Q, R and S graphical regions; which is the central and most highlighted portion of the ECG waveform. The detection of QRS-complex in ECG can be achieved by first three steps of DAF. In proposed algorithm, QRS detection is based on artificial intelligence approach [20,21,22], by considering the performance and effectiveness in Matlab IDE.

The blood circulation of human body can be used as an exclusive way to provide safe communication in BSN. The variation in time interval of heart beats is referred as heart rate variability (HRV). The authors in [2,5,8] have previously claimed that that HRV signals have exclusive properties and chaotic nature, which presents random characteristics and hence can be used for data authentication in BSN. Furthermore, contrasting conventional biometric cryptosystems in general networks such as fingerprint, facial pattern, palm print, hand geometry and iris pattern, the circulation of blood system in a human body produces a unique protected communication path particularly available for Body Sensor Networks (BSN).

The analysis of HRV includes the study of statistical indices such as standard deviation or other typical spectral analysis methods to observe fluctuations in heartbeats in ECG. Our conducted experimental system (including both hardware and software) results HRV measurement by using different approaches. There are two prominent methods to evaluate HRV, as studied by Lin *et al.* [23]. The first one is the evaluation of HRV in time domain by investigating the chain of RR intervals in ECG waveform. While, the second method takes in analysis of ECG in frequency domain, in which same spectrum of identical RR intervals is analyzed. The spectral analysis methods allow the decomposition of cardiovascular time series into its oscillatory elements, which offers non-invasive evaluation of the Autonomic Nervous System (ANS); the sympathetic and parasympathetic are its two main components. The heart rate is increased and decreased by sympathetic and parasympathetic, respectively. However, these methods (spectral analysis) symbolize a more complicated mode for examining time series of heart rate. Whereas, HRV measurement in time domain can reduce computational complexity and save more resources than frequency domain due to the fact that heart beats of ECG signals are recorded in time series. Therefore, time domain method of HRV calculation is focused in our research.

There are two important time domain approaches for HRV calculation, (i) SDNN (Standard Deviation of NN interval); and (ii) Root-Mean Squared of the Successive Differences (RMSSD). SDNN is based on calculation of standard deviation of consecutive NN intervals of heartbeats from start of the QRS-complex, where as NN interval is the time duration between consecutive R-R peaks. RMSSD can be calculated from the difference between consecutive NN intervals, as presented in Lin *et al.* [23]. The long-term recordings observed by time domain approaches must include in at least 18 h ECG recording. While performing experiment on 24 subjects, it was also observed that only SDNN and RMSSD were able to measure the cyclic components, which cause variations for both short-term and long-term recordings, *i.e.*, 5 min and 24 h, respectively. For that reason, we used SDNN-to-RMSSD ratio in our authentication protocol to make decision on whether to transmit data or not in BSN. If authentication protocol matches, then physiological information generated from sender could be transmitted to receiver, and if not, it is discarded. Besides, one of the reasons to exercise these two techniques collectively as authentication protocol is due to their ability of recording ECG in the short-term and long-term, because health monitoring in BSN is required for long durations. Another reason for SDNN-to-RMSSD ratio being feasible is because it demands less computation and unique statistical index, which can be utilized as a substitute for Low-Frequency to High-Frequency ratio (LH/HF) measured from the spectral analysis as elaborated in [24,25].

In this study, we employed SHA-1 as a low cost encryption technique to increase the level of authentication. Although DAF can provide authentication, to ensure further security, the least expensive and flexible encryption technique is used in the proposed algorithm, as shown in Figure 3. The SHA-1 stands for Secure Hash Algorithim-1 [26], producing 20 bytes or 40 hexadecimal digits hash value. It is very simple and cost-effective because it does not require high resources for encryption and decryption. Therefore, it is utilized in many applications and protocols, such as Secure Sockets Layer (SSL), Internet Protocol Security (IPSec), Secure Shell (SSH) and Transport Layer Security (TLS). The proposed algorithm provides unique, simple and efficient approach for securing BSN and its authentication is verified twice: (i) by using DAF; and (ii) by low cost encryption.

### 2.3. Data Authentication Function (DAF)

The DAF is utilized in proposed algorithm to provide efficient approach to secure BSN. It is the combination of five basic steps, which are discussed below:

Step#1: Linear Filtering

The linear filtering is based on differentiation, which is basically high-pass filtering process. It amplifies higher frequencies that are characteristic for the QRS—complex and attenuates lower frequencies that are characteristic for P and T deflections. Consider r[n] is the input raw ECG signal, and O[n] is the output of linear filtering, which is the linear combination of $d_{1}[n]$ and $d_{2}[n]$, first derivative and second derivative of r[n], respectively.

(1) $$d_{1}[n]=r[n]-r[n-2]$$

(2) $$d_{2}[n]=(r[n+2]-2\times r[n]+r[n-2])$$

(3) $$O[n]=d_{1}[n]+d_{2}[n]$$

Equation (1) represents first derivative $d_{1}[n]$ and Equation (2) demonstrates second derivative $d_{2}[n]$ of raw ECG signal r[n], whereas combination of both these derivative O[n] is shown in Equation (3).

Step#2: Non-Linear Transformations

In our proposed algorithm, the non-linear transformation is achieved by the combination of squaring operator and moving windows integration. The squaring operator s[n], as shown in Equation (4), gives optimal output by squaring O[n]. In QRS complex, it performs suppression if any difference arises due to P and T deflections, and also enhances amplitude of high frequency components. The moving window integration helps to make output from squaring operator further smooth. The moving window integration y(n) is calculated by using Equation (5)

(4) $$s[n]={\left\{O[n]\right\}}^{2}$$

(5) $$y(n)=\frac{1}{N}[s(n-(N-1))+s(n-(N-2))+..........+s(n)]$$

whereas N is the window width, which is equal to 67 and is appropriate at 128 Hz.

Step#3: Threshold Detection

The threshold detection in our algorithm is calculated by using Equation (6) and it is the essential step to discover the QRS-complex. For all value of i, where i is the number of heart beats or ECG signals involved in the ECG waveform, if outputs of non-linear transformation y[n] is greater than or equal to the predetermined threshold as represented in Equation (7), all these outputs are termed as QRS-complex.

(6) Thershold={max(y[n])−mean(y[n])}/2

(7) $$QRS_{i}=y{\left[n\right]}_{i}>=Thershold$$

Step#4: HRV Calculations

Once the QRS-complex is detected, the next step is to determine the R-Peak, which can further be used for HRV calculation. Equation (8) states that just by finding the absolute value or index of QRS-complex, we can determine R-Peak from the whole QRS-complex. In ECG, Q-to-Q intervals are the time intervals between successive heart beats during whole QRS-complex; they are also normally termed as RR intervals. We used Equation (9) to calculate RR interval in our research and the heart rate can be determined by Equation (10).

(8) R=|QRS|

(9) $$RR=RR_{i+1}-RR_{i}$$

Then, RR intervals are used to find heart rate variability (HRV) using two main time domain approaches SDNN and RMSSD. Both of these approaches have capability of long-term recording for 24-h; therefore in our study we measure HRV by both SDNN and RMSSD as shown in Equations (10) and (11).

(10) $$SDNN=\sqrt{\frac{1}{N}}\sum_{i=1}^{N}{\left(RR_{i}-\bar{RR}\right)}^{2}$$

whereas, $\bar{RR}=\frac{RR_{1}+RR_{2}+..........+RR_{N}}{N}$, RR, $\bar{RR}$, N represents R-to-R interval, mean of R-to-R interval and number of peaks of ECG waveform, respectively.

(11) $$RMSDD=\sqrt{\frac{1}{N-1}}\sum_{i=1}^{N}{\left(RR_{i}-RR_{i}\right)}^{2}$$

**Table 1:** The average Standard Deviation of NN interval (SDNN) and average Root-Mean Squared of the Successive Differences (RMSSD), and SDNN-to-RMSSD (SRR) for different subjects.

Subjects	Average SDNN	Average RMSSD	SDNN-to-RMSSD Ratio (SRR)	SRR*10^3^	SRR*10^3^ (Binary 16 Bits)
1	87.823	46.877	1.873	1873	0000011101010001
2	88.294	29.964	2.946	2946	0000101110000010
3	65.404	24.712	2.646	2646	0000101001010110
4	99.772	56.776	1.757	1757	0000011011011101
5	64.684	24.926	2.595	2595	0000101000100011
6	60.779	31.389	1.936	1936	0000011110010000
7	103.311	86.998	1.187	1187	0000010010100011
8	95.670	45.338	2.110	2110	0000100000111110
9	67.413	28.804	2.340	2340	0000100100100100
10	145.398	82.814	1.755	1755	0000011011011011
11	171.083	121.290	1.410	1410	0000010110000010
12	61.675	30.409	2.028	2028	0000011111101100
13	107.385	76.436	1.404	1404	0000010101111100
14	77.977	31.235	2.496	2496	0000100111000000
15	89.431	61.906	1.444	1444	0000010110100100
16	69.660	30.627	2.274	2274	0000100011100010
17	62.985	31.688	1.987	1987	0000011111000011
18	57.524	17.604	3.267	3267	0000110011000011
19	113.543	79.594	1.426	1426	0000010110010010
20	97.117	66.308	1.464	1464	0000010110111000
21	70.131	47.749	1.468	1468	0000010110111100
22	72.651	39.758	1.827	1827	0000011100100011
23	100.784	74.449	1.353	1353	0000010101001001
24	61.608	36.512	1.687	1687	0000011010010111

Step#5: Authentication Protocol

The ratio between SDNN and RMSSD, termed SRR, as shown in Equation (12), is used in authentication protocol of the proposed algorithm. According to Sollers *et al.* [25], SRR demands less computation. It is stated on basis of calculation from different contexts such as standing and sitting position of subjects and it is observed to be a good and reliable statistical index for both patients and normal health monitoring. The main advantages of using time domain estimated index are: (i) less computational complexity; and (ii) low alarm for stationary of the time series [24]. Therefore, because of all these benefits, SRR is utilized in our authentication protocol to reduce the computational complexity so as to provide efficient approach for securing BSN.

(12) SRR=SDNN/RMSSD

In our conducted experiment, it is observed that SRR is the unique biometric index. The SRR values for both short-term and long-term analysis are observed to be distinctive. Table 1 states that average SRR values (for 8 h) of 24 subjects are distinct. Therefore, it can be used to differentiate a person from any other during data transmission in BSN. Furthermore, SRR, along with gender and age of the subject (transmitter), are used to generate matchless authentication key.

**Figure 4:**
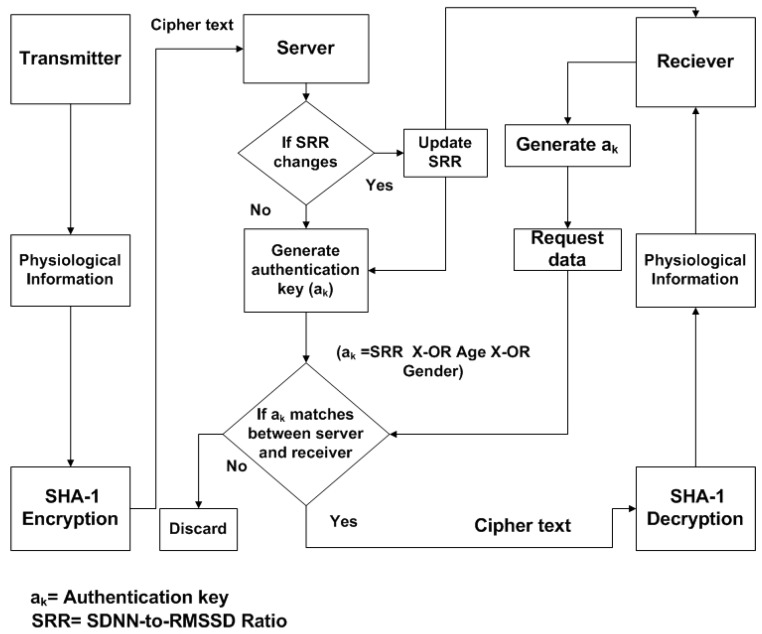
The block diagram of communication model for Body Sensor Networks (BSNs) using proposed authentication protocol.

The block diagram of the communication model for the proposed algorithm is shown in Figure 4. After collecting the physiological information from specific sensor node at transmitter side, low cost SHA-1 hashing encryption technique is applied to generate cipher text. The cipher text from transmitter is transferred to the remote sever; the inexpensive encryption is applied to original physiological information in order to provide secure communication between them. When receiver (surgeon) requests data from the sever, the authentication key (a_k_) will be checked. If a_k_ matches between server and receiver, the receiver will obtain the cipher text and decodes it by using SHA-1 to get back original bio-signal. Sever observes the SRR periodically, in case any change occurs in SRR, sever will update to the receiver. The receiver will generate the authentication key by using update SRR.

**Figure 5:**
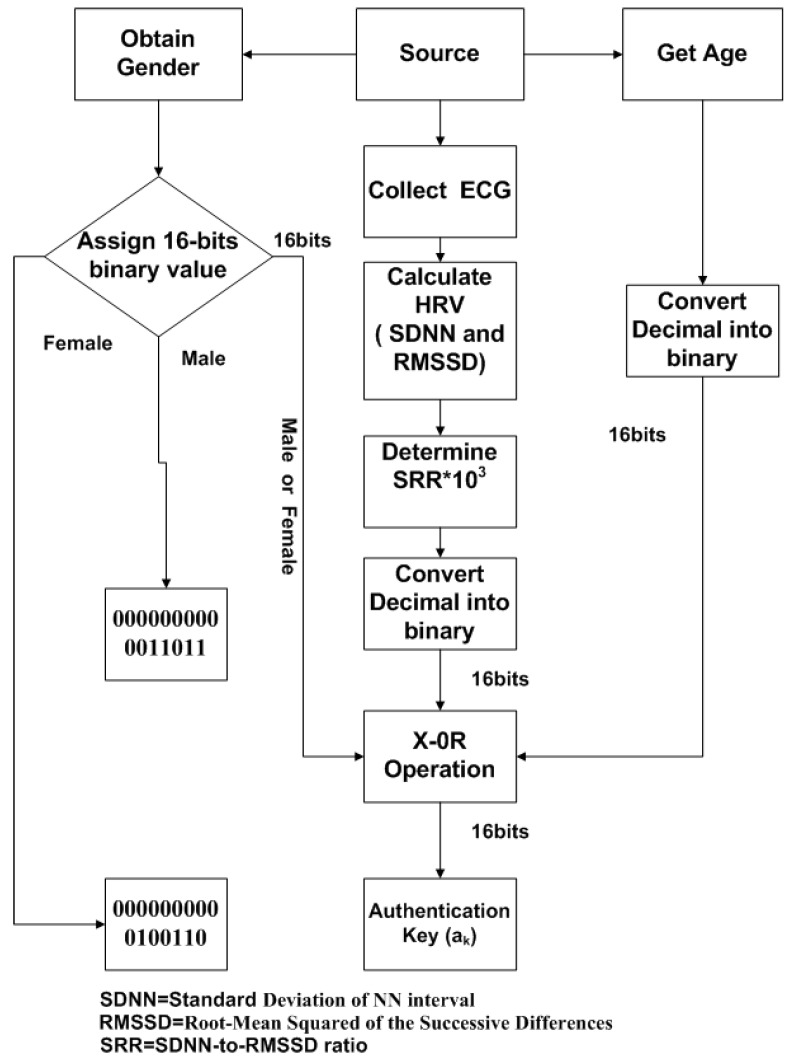
The key generation procedure for proposed algorithm.

The key generation procedure in our proposed algorithm is quite simple and efficient. It is based on X-OR logical operation, as shown is Figure 5. The ECG signal from source node is obtained and HRV is measured by using statistical indices SDNN and RMSSD. Moreover, SRR (SDNN to RMSSD ratio) is calculated, the SRR value is multiplied by 1000, or 1 k, to get a 4-digit decimal number, and converted into the equivalent 16-bit binary number. The authentication key (a_k_) is the logical X-OR between SRR, age and gender of the source (a_k_ = SRR (16 bits) X-OR age (16 bits) X-OR gender (16 bits)). The X-0R outputs “1” only when inputs have different value. The output of a_k_ will be a binary number (16 bits), which is acting as key between source and destination for data transmission in BSN. Table 1 suggests that SRR is the unique biometric index, along with that age and gender information of the subject is also used in order to generate robust authentication key.

Consider subject 1 transmits the physiological information, having a 16-bit SRR of 0000011101010001, as shown in Table 1. The subject is male and his age is 29 years. To obtain a 16-bit equivalent value for “male”, assign 0 to 25 decimal numbers for alphabet “a” to “z”, respectively, therefore for “male” subject, the 16-bit binary number is 0000000000011011 (12 + 0 + 11 + 4 = 27). The age of the subject is 29; the binary equivalent of 29 is 0000000000011101 (16 bits). The authentication key, a_k_, is the output of X-OR operation between these three 16-bits binary numbers (0000011101010001, 0000000000011011 and 0000000000011101)_,_ after applying the X-OR operation, the results will be 0000011101010111 (16 bits). The 16 bits output of X-OR will be authentication key between server and receiver.

The statistical indices such as SDNN and RMSSD for measurement of HRV satisfy all properties of ideal biometric traits [2]. The ideal biometric properties are universal, unique, everlasting or permanent, measurable, efficient, adequate and reliable or unassailable. The HRV is universal as it can be measured for all subjects and acceptable to patient as well as organization to utilize as an identifier (adequate). The uniqueness of SRR can be verified from Table 1, as all 24 subjects have different SRR. According to [25], SRR is not complex (easily measureable), good (efficient) and reliable (quite complicated to recreate by fake acts). The SRR is permanent for a reasonable period of time, because it is observed during conducted experiment that HRV of a patient does not change significantly with respect to time matching criteria over a reasonable period of time.

In [27], it is stated that mental stress level of the person could cause a change in HRV. Therefore, in the proposed algorithm, SRR is observed periodically by a server. On the one hand, to check SRR periodically consumes some extra memory, but still the proposed algorithm provides efficient method for data authentication in BSN. On the other hand, the varying nature of SRR due to mental stress condition could increase the strength of security, because authentication key keeps varying in accordance to change in SRR. In case one authentication key is compromised (still cannot receive original physiological information because data is converted into cipher text by using SHA-I encryption) hacker cannot receive all cipher texts of physiological information of the subject because authentication key continues changing according to SRR. The performance of the proposed algorithm is analyzed in next section.

### 2.4. Performance Analysis

Our proposed algorithm utilizes simple key generation procedure based on SRR to secure data processing in BSN. This advantage leads our algorithm toward simplicity in comparison with all other available approaches for securing BSN, in view of the fact that complex key production processes make existing methods more complex and ineffective than proposed algorithm.

When conventional schemes, such as symmetric encryption approaches, are used to apply security in BSN by utilizing external key, only single key is required for both encryption and decryption. There are two main reasons, which cause high transmission time and more resource utilization with these approaches. Firstly, unique keys are generated for different rounds from initial key such as in DES original key size is 64 bits, from which 8 different keys of size 56 bits are generated. Secondly, they support only fixed block size of data, such as 64 bits for DES. If data size is more than 64 bits, it must be divided into multiple blocks of 64 bits and data is transmitted in multiple rounds.

While utilizing asymmetric encryption approach for implementing security, even more transmission time and resources are consumed due to the complex process for key generation and data transmission. RSA is one of the examples of public-key based encryption, which uses two different keys during data transmission, public and private keys for encryption and decryption, respectively. Nevertheless, it requires more resources for data transmission than the proposed algorithm, even more than DES. When physiological features are used for key generation, such as in PSKA [14], they do reduce the transmission time compared with utilization of external keys, but their complex key generation procedure makes them cost inefficient solutions for securing BSN.

**Figure 6:**
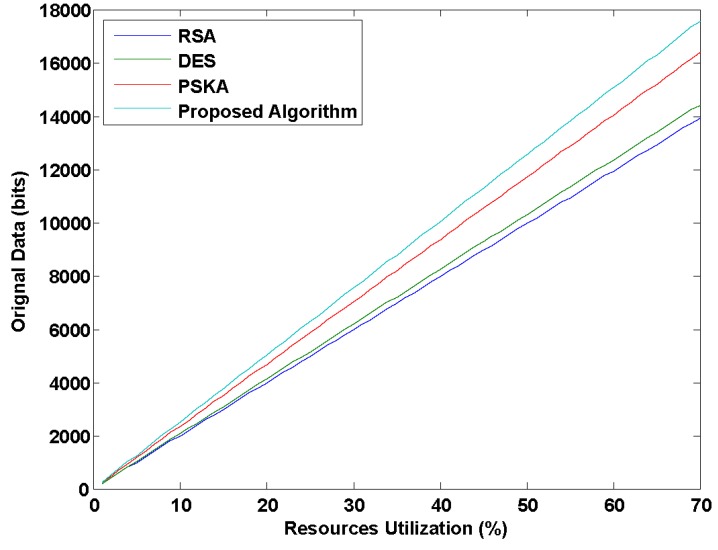
Amount of data transmission in bits with same percentage of resource utilization for different methods.

Figure 6 explains that our proposed algorithm can transmit a higher number of bits than PSKA, DES and RSA by using same number of resources due to the use of a simple and efficient key generation mechanism in our proposed algorithm. As complex key generation procedures waste more resources, our proposed algorithm eliminates the need of time consuming and inefficient methods for data authentication in BSN, resulting in low utilization of resources. Hence, it can be stated that our proposed algorithm is a cost effective approach for securing BSN.

To explain the efficiency of our proposed algorithm, a simulation environment with the real-time data of 20 subjects was created and parameters used in performance analysis are shown in Table 2. To analyze the performance of our proposed algorithm, we used real-time ECG data of 20-year-old female subjects. The length of this ECG data is 258 decimal values in different matrix formats, and each decimal value is equal to 36 bits, which means overall length in binary is 9216 bits. If authentication protocol matches between source and destination, then all 9216 bits will be transmitted in one round. On the contrary, this is not case for DES and RSA, because DES only supports data size of 64 bits and in RSA message of only one decimal (*i.e.*, 36 bits) value can be transmitted at a time. Although PSKA will send the complete information of 9216 bits, the complexity involved in its algorithm requires more processing time than proposed algorithm.

**Table 2:** List of abbreviations used in performance analysis.

Abbreviation	Detail	Abbreviation	Detail
t	Total simulation time	$L_{b}$	Data length in binary
k	Complexity per round	$E_{i}$	Initial energy
$N_{r}$	Number of rounds	$E_{ar}$	Average remaining energy
$N_{k}$	Number of keys required for all rounds	$E_{rd}$	Remaining energy for destination
$K_{r}$	Number of keys required per round	$E_{rs}$	Remaining energy from source
$K_{i}$	Initial key size	$T_{x}$	Transmission power
$L_{d}$	Data length in decimal	$R_{x}$	Reception power
D	Data rate	$P_{u}$	Power utilized

#### 2.4.1. Transmission Time

The transmission time is the amount of time required for complete data transmission from source to destination. It depends upon the number of rounds involved during transmission. As the number of rounds increases, transmission time will increase, as shown in Equation (13).

(13) $$t=N_{r}\times k$$

here, t represents total transmission time, $N_{r}$ demonstrates number of rounds, and k denotes the complexity involved per round. For our proposed algorithm, $N_{r}=1$ because all data is transmitted in one round, provided that the authentication key (a_k_) matches between source and destination. As the complex procedure for the construction of the key is omitted, the proposed algorithm requires less transmission time for data transmission in BSN. While for PSKA, $N_{r}=1$ but t also depends on k, as the value of k in PSKA is greater than it is in the proposed algorithm, caused by the fact that PSKA is based on complex procedure for key generation. As a result, PSKA takes more time for processing of entire information from source to destination than proposed algorithm does.

For DES, the number of rounds depends upon the initial key size and length of data generated from the source. The number of rounds is directly proportional to the length of data and inversely proportional to initial key size. In Equation (14), $L_{b}$ and $K_{i}$ represent length of data in bits and initial key size in bits, respectively.

(14) $$N_{r}=\frac{L_{b}}{K_{i}}$$

In our simulation, $L_{b}$ is 9216 bits and $K_{i}$ is 64 bits for DES, therefore $N_{r}$ will be 144 rounds:

$$N_{r}=\frac{L_{b}}{K_{i}}=\frac{9216}{64}=144$$

The overall number of keys required for all rounds $N_{k}$ depends upon $N_{r}$ and keys required per round $K_{r}$, as shown in Equation (15).

(15) $$N_{k}=N_{r}\times K_{r}$$

$$N_{k}=144\times 8=1152$$

It can be visualized that DES uses 144 rounds and 1152 keys in order to achieve authentication. In addition, this complex calculation takes a longer transmission time, compared to our proposed algorithm. While for RSA, the number of rounds ($N_{r}$) is equal to the length of data in decimal ($L_{d}$). In our simulation $L_{d}$ is 258, so $N_{r}$ is also 258. Since transmission time depends upon number of rounds and complexity involved per round, in both conditions RSA requires more time to process complete data than our proposed algorithm and DES, evident from the following mathematical values. For our proposed algorithm, DES, and RSA, $N_{r}=1$, $N_{r}=144$ and $N_{r}=258$, respectively.

#### 2.4.2. Average Remaining Energy

Let, $E_{i}$ be the initial energy of source and destination for data transmission. The amount of energy consumed is proportional to the transmission time. The larger the time required for processing, the more energy will be consumed. To check whether or not our proposed algorithm is energy efficient, it is necessary to determine the average remaining energy. Equation (18) calculates average remaining energy of both source and destination. $E_{rs}$ and $E_{rd}$ represent remaining energy of source and destination, respectively, depending upon the data length ($L_{d}$), data rate (D) and transmitting/receiving power ($T_{x}/R_{x}$), as shown in Equations (16) and (17).

(16) $$E_{rs}=\sum_{j=1}^{N}[{\left(E_{i}\right)}_{\left(j-1\right)}-(T_{x}\times L_{d}/D)]$$

(17) $$E_{rd}=\sum_{j=1}^{N}[{\left(E_{i}\right)}_{\left(j-1\right)}-(R_{x}\times L_{d}/D)]$$

(18) $$E_{ar}=(E_{rs}+E_{rd})/2$$

The value of $E_{ar}$ is high for our proposed algorithm, as explained in Table 3, because it requires less transmission time. Hence, it can be believed that the proposed algorithm is simple, as it consumes less time in processing of complete information, ultimately consuming less energy than PSKA, DES and RSA.

#### 2.4.3. Total Power Required

Our proposed algorithm is also power efficient. As utilization of power depends upon energy consumption and transmission time, as explained in Section 2.4.1 and Section 2.4.2, since our proposed algorithm utilizes less energy and time for data transmission, it can be stated that less power will be utilized for the proposed algorithm to secure BSN. The amount of power utilized ($P_{u}$) can be measured by Equation (19), depending upon the initial energy ($E_{i}$), data rate (D), data length ($L_{d}$), total transmission time (t), and transmitting/receiving power ($T_{x}/R_{x}$).

(19) $$P_{u}=\frac{1}{t}\sum_{i=1}^{N}\left[2\times E_{\left(i-1\right)}-\frac{L_{d}}{D}(T_{x}+R_{x})\right]$$

The comparison on basis of transmission time required, average remaining energy and power utilization between the proposed algorithm, PSKA, DES, and RSA is shown in Table 3. The detail discussion of Table 3 is provided in Section 4.

**Table 3:** Comparison between proposed algorithm, Physiological Signal based Key Agreement (PSKA), Data Encryption Standard (DES) and Rivest Shamir Adleman (RSA).

Methods	Parameters		
	Transmission Time (ms)	Average Remaining Energy (Joules)	Power Utilization (mW)
Proposed Algorithm	0.207	0.998	9.64
PSKA	0.239	0.976	9.89
DES(Symmetric Encryption Approach)	3.40	0.963	10.05
RSA(Asymmetric-Encryption Approach)	6.40	0.932	10.10

## 3. Experiment Setup

In our conducted experiment, 24 healthy, right-handed subjects (all male ranging from 20 to 28 years of age, with no history of cardiovascular disease) were recruited from a local university to participate in this pilot study. Informed written consent was provided by all participants before completing questionnaires or undergoing physiological assessment. All subjects were tested under standard conditions between 1:00 p.m. and 5:00 p.m., at a room temperature of 22 °C–26 °C after abstaining from smoking and coffee consumption for 6 h before participation in the experiment. They were asked to wear the device comfortably with regular electrodes. Lead II ECG signals and respiration signal were collected for each subject simultaneously. In this pilot study, HRV temporal parameters included: (i) SDNN, which is a global index of HRV and reflects all long-term components and circadian rhythms responsible for variability in the recording period; (ii) RMSSD, which reflects parasympathetic nerve activity; and (iii) HRV triangular index (HRV TI), which serves as an estimate of the overall HRV.

It is observed during the experiment that two time domain approaches namely SDNN and RMSSD were able to record for a long duration, in addition their calculation is simple and not time consuming. As in BSN, it is necessary to monitor the patient for the whole day (24 h). Therefore, in this scenario, these two techniques are best suited to measuring HRV for further simulation to provide proficient method for data authentication in BSN.

## 4. Simulation and Results

Matlab is used in the simulation. It is assumed that initial energy is one joule for both source and destination. Transmitting power, receiving power and data rate are considered −25 dbm, −95 dbm and 256 Kbps, respectively. A comparative analysis of proposed algorithm, PSKA technique and two other conventional cryptographic authentication-based approaches, Data Encryption Standard (DES) and Rivest Shamir Adleman (RSA), is discussed in this section. We used publicly available source for simulation, the real time data of ECG from physioNet including two databases, MIT-BIH Normal Sinus Rhythm Database and MIT-BIH long-Term ECG Database. Overall, we analyzed ECG data of 20 patients between the ages of 20 and 80.

**Figure 7:**
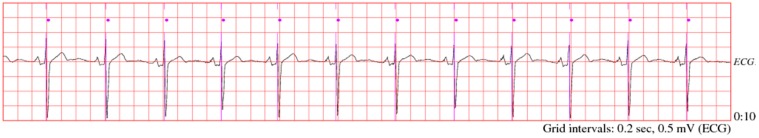
ECG waveform of 20-year-old female patient.

**Figure 8:**
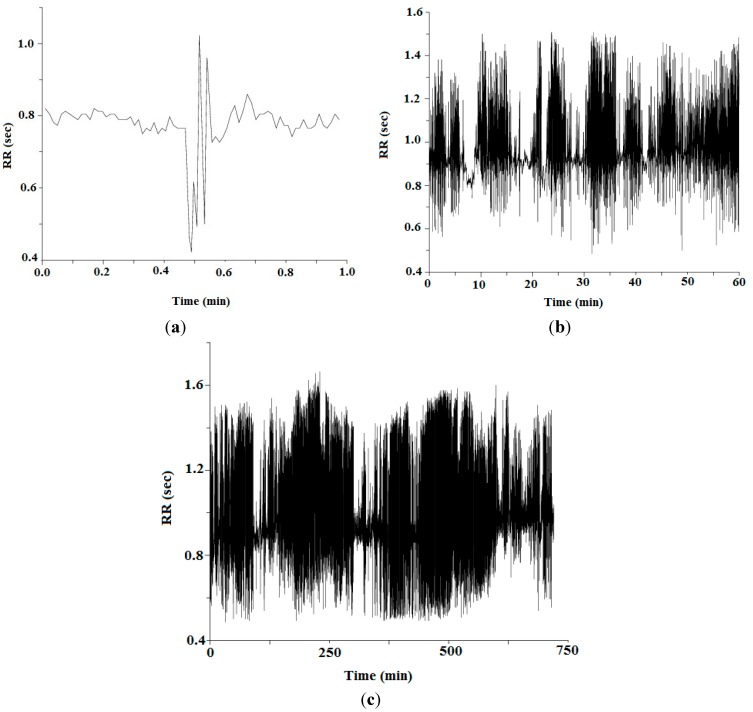
The inter-beat (RR) interval representation (**a**) RR-interval for 1 min; (**b**) RR-interval for 1 h; and (**c**) RR-interval for complete wave.

**Figure 9:**
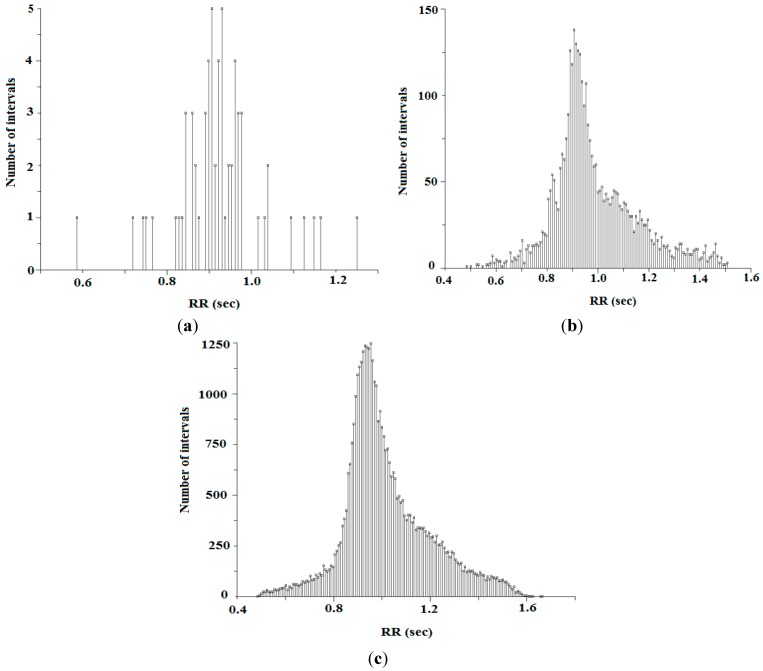
(**a**) Histogram representation of RR-interval for 1 min; (**b**) histogram representation of RR-interval for 1 h; and (**c**) histogram representation of RR-interval for complete wave.

Figure 7 represents ECG waveform of a 20-year-old subject (female) for duration of 10 s. The grid interval used in this waveform is 0.2 s and amplitude is 0.5 mV. A 12-bit Analog-to-Digital converter sampling at 128 Hz frequency is used to get the digital signal. The RR-interval representation at different time durations of the mentioned subject is shown in Figure 8. This time interval is distance between two consecutive R-peaks. Figure 8a–c exploits the RR-interval for time duration of 1 min, 1 h and for complete wave, respectively. Figure 9 demonstrates histogram of RR-interval for different time durations. Figure 9a–c explains histogram for RR-interval representation for time duration of 1 min, 1 h and for complete wave, respectively.

Figure 8 and Figure 9 represent recording of RR-intervals for both the short-term and long-term. The RR-intervals are further used to measure HRV at different time duration by using both time domain approaches SDNN and RMSSD, as shown in Equations (10) and (11), respectively. However, due to the ability of long-term recording, these two techniques are preferred, because in a real scenario, health monitoring of patients should be observed for 24 h.

**Figure 10:**
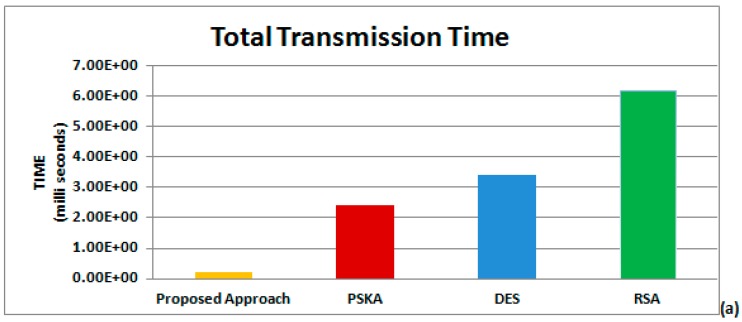

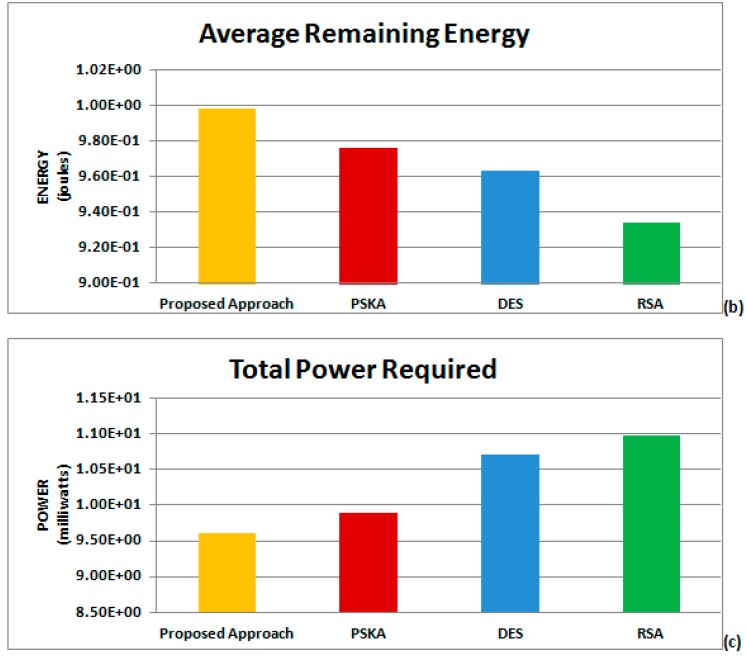
Performance analysis: (**a**) total Transmission Time for different methods; (**b**) average remaining energy for different methods; and (**c**) total power required for different methods.

The ratio between SDNN and RMSSD (SRR) is further used in authentication protocol to generate authentication key (a_k_) between source and destination to provide efficient solutions to secure BSN, as shown in Figure 10a–c. The a_k_ in our proposed algorithm is based on simple, cost-effective and proficient key generation procedure, as shown in Figure 5. Table 3 demonstrates that the proposed algorithm is more efficient than PSKA, RSA and DES. Figure 10a shows that the total amount of transmission time required for the proposed algorithm, PSKA, DES and RSA is 0.207 ms, 0.239 ms, 3.40 ms and 6.40 ms, respectively. From the results, the detailed analysis shows that the proposed algorithm consumes less time than other techniques during transmission. In addition to more transmission time consumption, the key generation procedure for these compared techniques also consumes more resources, which makes all compared methods not cost-effective solution for implementing security in BSN.

Figure 10b reveals the average remaining energy of our proposed algorithm, PSKA, DES and RSA as 0.998 J, 0.976 J, 0.963 J and 0.932 J, respectively. As the implementation of the proposed algorithm is simpler and easier than PSKA, DES and RSA, it requires less time and energy than all other compared techniques. Total power consumed by the proposed algorithm, PSKA, DES and RSA during data transmission is 9.64 mW, 9.89 mW, 10.05 mW and 10.10 mW, respectively, as shown in Figure 10c. It can be clearly observed that our proposed algorithm is more power-efficient than PSKA, DES, and RSA.

## 5. Conclusions

This paper presents a novel algorithm based on Heart Rate Variability (HRV) to attain security and privacy during physiological information processing in BSN, whereas ECG is used as a biometric feature. Our research aims to illustrate the deployment of HRV to provide efficient method for securing Body Sensor Networks (BSN). However, no existing work has used the ratio between SDNN and RMSDD to secure BSN. An experiment on 24 healthy subjects was conducted in our laboratory to take out ECG signal from a textile electrode and HRV is measured using several approaches. Along with this, real-time data of ECG from physiobank is used in a simulation. We utilized two databases, namely MIT-BIH Normal Sinus Rhythm Database and MIT-BIH long-Term ECG Database, to carry out experiments. The real-time ECG data of 20 patients between 20 and 80 years old is received from both databases. We used Data Authentication Function (DAF) in our proposed algorithm to secure BSN, instead of using complex procedure for keys production. The DAF involves five major steps: (i) linear filtering of ECG signal to pass high frequency of QRS-complex and attenuates low frequency of P and T regions of ECG; (ii) non-linear transformation is applied to achieve further smoothness in QRS detection; (iii) threshold detection, to find R-peak of ECG signal; (iv) to calculate Heart Rate Variability (HRV) by using different time-domain and frequency-domain approaches; and (v) SRR, ratio between Standard Deviation of NN-interval (SDNN) and Root-Mean Squared of the Successive differences (RMSSD) is used in authentication protocol. However, BSN health monitoring is required over a long time, therefore these time-domain approaches are preferred due to their ability of recoding ECG for long duration and also their computation is not complex. The 16-bit binary number acts as authentication key between source and destination in our research paper, which is produced by a simple key generation process utilizing SRR, age and gender information of the source. Transmission will only begin if authentication key matches between server and receiver, otherwise message will be discarded. Simulation results show that our proposed scheme outperforms all three compared techniques, PSKA, DES and RSA. It can be concluded by simulation results that our proposed algorithm requires less transmission time than PSKA, DES and RSA; *i.e*., 0.207 ms, 0.239 ms, 3.40 ms and 6.40 ms, respectively. Average remaining energy of our proposed algorithm, PSKA, DES and RSA is observed as 0.998 J, 0.976 joules 0.963 joules and 0.932 joules, respectively. Total power required by our proposed algorithm is 9.64 mW, which is lower than power consumption of PSKA, DES and RSA, which are 9.89 mW, 10.05 mW and 10.10 mW, respectively. As fewer resources are required during data transmission, our proposed algorithm also claims to offer a more cost-efficient solution than conventional approaches for data authentication in BSN.

The future work is to perform the experiment with an increased number of nodes and to modify authentication protocol in order to provide even more efficient method for data authentication in BSN. Moreover, to calculate the Half Total Error Rate (HTER) of our proposed algorithm and to compare with other existing techniques is also a future task. In addition, a BSN platform, on the basis of the proposed algorithm, will be implemented in order to verify simulation results.
