# Erratum: Sandeep P., et al. An Efficient Biometric-Based Algorithm Using Heart Rate Variability for Securing Body Sensor Networks. *Sensors* 2015, *15*, 15067–15089

**DOI:** 10.3390/s17030607

**Published:** 2017-03-16

**Authors:** Sandeep Pirbhulal, Heye Zhang, Subhas Chandra Mukhopadhyay, Chunyue Li, Yumei Wang, Guanglin Li, Wanqing Wu, Yuan-Ting Zhang

**Affiliations:** 1 Shenzhen Institutes of Advanced Technology, Chinese Academy of Sciences, Shenzhen 518055, China; sandeep@siat.ac.cn (S.P.); hy.zhang@siat.ac.cn (H.Z.); cy.li1@siat.ac.cn (C.L.); gl.li@siat.ac.cn (G.L.); ytzhang@ee.cuhk.edu.hk (Y.-T.Z.); 2 Key Laboratory for Health Informatics of the Chinese Academy of Sciences (HICAS), Shenzhen Institutes of Advanced Technology, 1068 Xueyuan Avenue, Shenzhen University Town, Shenzhen 518055, China; 3 Shenzhen College of Advanced Technology, University of Chinese Academy of Sciences, Shenzhen 518055, China; 4 School of Engineering and Advanced Technology, Massey University, Palmerston North 4442, New Zealand; S.C.Mukhopadhyay@massey.ac.nz; 5 Shenzhen Nanshan District Xili Hospital, Shenzhen 518055, China; fox_gxh@sina.com; 6 Key Laboratory of Human-Machine-Intelligence Synergic System, Shenzhen Institutes of Advanced Technology, Chinese Academy of Sciences (CAS), Shenzhen 518055, China; 7 Joint Research Centre for Biomedical Engineering, Chinese University of Hong Kong, Shatin N.T., Hong Kong, China

The authors wish to make the following corrections to their paper [1]:

1. The first affiliation of the authors was incorrect in the published paper. Therefore, it is corrected from “Institute of Biomedical and Health Engineering, Shenzhen Institutes of Advanced Technology, Shenzhen 518055, China” to “Shenzhen Institutes of Advanced Technology, Chinese Academy of Sciences, Shenzhen 518055, China”.
2. One affiliation of the author Sandeep Pirbhulal was not included in the published paper. Therefore, Sandeep Pirbhulal’s affiliation namely “Shenzhen College of Advanced Technology, University of Chinese Academy of Sciences, Shenzhen 518055, China” has now been added.

The authors would like to apologize for any inconvenience caused to the readers by these changes. The manuscript will be updated and the original will remain online on the article webpage.

## Supplementary Materials

Supplementary File 1
